# Suppression of AGTR1 Induces Cellular Senescence in Hepatocellular Carcinoma Through Inactivating ERK Signaling

**DOI:** 10.3389/fbioe.2022.929979

**Published:** 2022-07-13

**Authors:** Houhong Wang, Yayun Cui, Huihui Gong, Jianguo Xu, Shuqin Huang, Amao Tang

**Affiliations:** ^1^ Department of General Surgery, The Affiliated Bozhou Hospital of Anhui Medical University, Bozhou, China; ^2^ Department of Cancer Radiotherapy, The First Affiliated Hospital of USTC, Division of Life Sciences and Medicine, (Anhui Provincial Cancer Hospital), University of Science and Technology of China, Hefei, China; ^3^ Faculty of Health and Life Sciences, Oxford Brookes University, Oxford, United Kingdom; ^4^ Department of Gastroenterology, The Affiliated Hangzhou First People’s Hospital, Zhejiang University School of Medicine, Hangzhou, China

**Keywords:** AGTR1, hepatocellular carcinoma, cellular senescence, sorafenib, ERK signaling

## Abstract

**Objective:** Cellular senescence is an effective barrier against tumorigenesis. Hence, it is of significance to characterize key features of cellular senescence and the induction of senescence in hepatocellular carcinoma (HCC) cells via pharmacological interventions. Our study determined the biological roles as well as mechanisms of angiotensin II type I receptor (AGTR1) on cellular senescence in HCC.

**Methods:** Lentivirus vector-mediated overexpression or knockdown of AGTR1 was conducted in HCC cells, respectively. A volume of 8 μM sorafenib was used to induce cellular senescence, and ERK was activated by 30 ng/ml ERK agonist EGF. Proliferation was evaluated *via* clone formation assay. HCC cell senescence was examined by flow cytometry for cell cycle, senescence-associated β-galactosidase (SA-β-gal) staining, and senescence-associated heterochromatin foci (SAHF) analysis. AGTR1, p53, p21, extracellular signal-regulated kinase (ERK), and p-ERK expression were assessed through Western blot or immunofluorescence.

**Results:** AGTR1-knockout HCC cells displayed the attenuated proliferative capacity, G2-M phase arrest, increased expression of p53 and p21, and elevated percentages of SA-β-gal- and SAHF-positive cells. In sorafenib-exposed HCC cells, overexpressed AGTR1 enhanced the proliferative capacity and alleviated G2-M phase arrest as well as decreased p53 and p21 expression and the proportions of SA-β-gal- and SAHF-positive cells. Moreover, AGTR1 knockdown attenuated the activity of p-ERK in HCC cells, and ERK agonist ameliorated AGTR1 knockdown-induced cellular senescence.

**Conclusion:** This study demonstrates that suppression of AGTR1 induces cellular senescence in HCC through inactivating ERK signaling. The significant synergistic effect of AGTR1 suppression and sorafenib might represent a potential combination therapy for HCC.

## Introduction

Liver cancer remains a global health challenge, with an estimated incidence of more than one million cases by 2025 (Li et al., 2022). Hepatocellular carcinoma (HCC) represents the dominating type of liver cancer, occupying ∼90% of all cases (Vogel et al., 2021; Cheng et al., 2022). Due to late diagnosis and damaged liver function, the five-year survival rate remains about 15% (Galle et al., 2021). Sorafenib is a multiple-target tyrosine kinase inhibitor, which has been approved as the only first-line therapeutic targeted drug against advanced HCC (Benson et al., 2021). It inhibits HCC cell proliferation via inactivating Ras/Raf/MEK/ERK signaling as well as targets PDGFR-β, VEGFR2, and c-KIT, etc., to weaken angiogenesis (Sun et al., 2021). Regrettably, only about 30% of HCC patients benefit from sorafenib and often typically experience resistance within 6 months (Finn et al., 2020; Qin et al., 2021). Additionally, some patients cannot tolerate sorafenib toxicity. Hence, it is imperative to discover more effective combination therapies to enhance the sensitivity of HCC cells to sorafenib as well as improve the efficacy.

Cellular senescence is a key process that modulates distinct pathophysiological processes from embryonic development to aging (Ziegler et al., 2021), which is not just a cell arrest but rather an active mechanism regulating cellular homeostasis, fibrotic process, and microenvironment through hindering the proliferation of abnormal cells (Duan et al., 2022). Evidence demonstrates that cellular senescence prevents tumorigenesis via suppressing the proliferation of tumor cells (Chini et al., 2020). Additionally, senescent cells that express markers (p53 and p21, etc.) result in tissue repair via secreting the senescence-associated secretory phenotype (SASP) (Chini et al., 2020). Recently, inducing cellular senescence as a tumor suppressor mechanism has emerged as a promising strategy to suppress tumor growth (Prasanna et al., 2021). Several intrinsic and extrinsic inducers of cellular senescence have been discovered such as oncogene activation and chemotherapeutics (sorafenib, etc.) (Alimirah et al., 2020). Angiotensin II type I receptor (AGTR1) is a potent vasopressor hormone and the main regulator of aldosterone secretion, which is a key effector in controlling blood pressure and volume in the cardiovascular system (Qiao et al., 2021). Previous evidence demonstrates that AGTR1 exerts a crucial role in facilitating cancer progression. AGTR1 triggers ovarian cancer spheroid formation and metastases through upregulating lipid desaturation as well as suppressing endoplasmic reticulum stress (Zhang Q et al., 2019). Overexpressed AGTR1 defines a subset of breast cancer and may confer sensitivity to AGTR1 antagonist losartan (Rhodes et al., 2009). Also, it facilitates lymph node metastases of breast cancer via activating C-X-C chemokine receptor type 4 (CXCR4)/stromal cell-derived factor-1α (SDF-1α) as well as triggering cellular migration and invasion (Ma et al., 2019). Targeting AGTR1 attenuates oncogenicity of glioblastoma via disrupting NF-κB/CXCR4 signaling (Singh et al., 2020). Telmisartan, an AGTR1 inhibitor, triggers melanoma cell apoptosis and can synergize with vemurafenib through affecting cell bioenergetics (Jelena et al., 2019). Moreover, AGTR1 blocker candesartan attenuates proliferation and fibrosis in gastric cancer (Okazaki et al., 2014). Suppression of AGTR1 inhibits cell growth and invasion in pancreatic cancer (Guo et al., 2015). AGTR1 results in intratumoral immunosuppression through inducing PD-L1 expression in non-small cell lung carcinoma (Zhu et al., 2019; Yang et al., 2020). Although AGTR1 has been recognized as an oncogene in several cancer types, the role in HCC remains to be fully explored. Furthermore, the underlying mechanisms of its upregulation in HCC are unclear and required to be further elucidated.

In the present study, we sought to determine the biological roles and mechanisms of AGTR1 on cellular senescence in HCC. We first demonstrated the functions of AGTR1 in attenuating HCC cell senescence through ERK signaling, providing a novel potential target as well as a potential combination therapy of AGTR1 suppression with sorafenib for HCC.

## Materials and Methods

### Cell Culture and Administration

Human normal hepatocyte line L-02 (Chinese Academy of Sciences) and HCC cell lines HepG2 and Huh7 (Chinese Academy of Sciences) were cultured in Roswell Park Memorial Institute Media 1640 (RPMI-1640) (Gibco, United States) with 10% fetal bovine serum (FBS; Gibco) and 1% penicillin–streptomycin and incubated at 37°C in a humidified incubator with 5% CO_2_. To induce cell senescence by sorafenib (TargetMol, China), HCC cells were exposed to 8 μM sorafenib and grown in RPMI-1640 plus 10% FBS lasting 4 days. HCC cells were exposed to 30 ng/ml ERK agonist EGF (PeproTech, United States) to activate ERK.

### Virus Production and Target Cell Transduction

Human HEK293T cells (Chinese Academy of Sciences) were seeded onto a six-well plate. When the confluence was 80%, the cells were transfected with 2 μg lentiviral vector that expressed full-length human AGTR1 or short hairpin RNA (shRNA) lentivirus vector of AGTR1 (GenePharma, China) utilizing Lipofectamine 3000 (Invitrogen, United States). Afterward, the cells were maintained in RPMI-1640 with 10% FBS. The supernatant with lentivirus was collected at 48 h. HCC cells were maintained in RPMI-1640, followed by a mixture of 2 × 10^5^ cells with 450 μl lentivirus-containing supernatants along with 4 μg/ml polybrene (Sigma-Aldrich, United States). Afterward, they were seeded onto a six-well plate, with replacement of RPMI-1640 after half an hour. After 2 days, the transduced cells were planted onto a 10-cm culture dish plus 4 μg/ml puromycin. Finally, we harvested the stably transduced cells.

### Western Blot

HCC cells were collected in a 1.5-ml centrifuge tube and centrifuged at 1500 rpm for 5 min. After removal of the supernatant, an appropriate amount of cell lysis buffer (Takara, China) was added containing PMSF, protease inhibitor, and phosphatase inhibitor. Then, the cells were lysed on ice for 30 min and then sonicated for 5 s. After centrifugation at 12,000 rpm for 10 min at 4°C, the supernatant was transferred to a freshly labeled 1.5-ml centrifuge tube. BCA kit was applied for quantification, and the remaining protein was added with 1/4 of the sample volume of 5× protein loading buffer. After blowing using a pipette, they were placed in a metal bath heater and heated at 100°C for 10 min. For denaturation, the denatured samples can be stored in a −80°C freezer. The protein was separated with sodium dodecyl sulfate-polyacrylamide gel electrophoresis (SDS-PAGE), along with transference onto a polyvinylidene difluoride membrane (Millipore, United States). The membrane was sealed by 5% skim milk, along with incubation with primary antibody of AGTR1 (1:500; 25343-1-AP; Proteintech, China), p53 (1:5000; 60283-2-Ig; Proteintech), p21 (1:3000; 10355-1-AP; Proteintech), p-p53 (1:4000; 28961-1-AP; Proteintech), p-p21 (1:1000; ab47300; Abcam, United States), ERK (1:1000; 51068-1-AP; Proteintech), p-ERK (1:1000; 28733-1-AP; Proteintech), or GAPDH (1:5000; HRP-60004; Proteintech). Being incubated with secondary antibodies, signals were visualized using ECL reagents (Takara). The gray value was quantified with ImageJ software.

### Clone Formation Assay

HCC cells were inoculated onto a six-well plate (600 cells per well) with a culture medium for two weeks. Afterward, the clones were fixed with 4% paraformaldehyde (Sangon Biotech, China) as well as dyed with crystal violet (Sigma-Aldrich). The number of clones (≥ 50 cells) was counted microscopically.

### Flow Cytometry

HCC cells were digested with 0.25% trypsin, along with centrifugation at 800 rpm lasting 5 min to prepare a cell suspension. The cell suspension was inoculated onto a six-well plate (20,000 cells per well) lasting 8 h. After waiting for the cells to adhere, the serum-free culture was continued for 12 h. After processing as intended, the cells were harvested into 15-ml centrifuge tubes. The supernatants were removed, along with being washed twice utilizing pre-chilled PBS. Afterward, digestion and centrifugation at 1200 rpm lasting 5 min were carried out. Being washed with PBS to remove cellular debris and residual impurities, the prepared pre-cooled 70% alcohol was added to the cells and placed at 4°C overnight to achieve the immobilization effect. The fixed cells were centrifuged and washed with PBS to remove residual alcohol. The PI staining solution was added to the cells, and the cell cycle was measured through flow cytometry (BD, United States) within 30 min after staining in the dark.

### Senescence-Associated β-Galactosidase Staining

HCC cells were inoculated onto a six-well plate and fixed with 4% paraformaldehyde for 5 min at room temperature. Being washed with PBS, the cells were incubated with fresh SA-β-gal staining reagent (Cell Biolabs, United States) supplemented with 1.0 mg/ml X-galactosidase at 37°C lasting 18 h for visualizing SA-β-gal staining. The senescent cells were photographed under a microscope. The SA-β-gal-positive percentage was quantified with ImageJ software.

### Senescence-Associated Heterochromatin Foci Assay

SAHF are specialized domains of facultative heterochromatin senescent cells. HCC cells were inoculated onto a 15-mm confocal dish (2 × 10^4^ cells per well). The cells were fixed in 4% paraformaldehyde, and dyed utilizing 4’,6-diamidino-2-phenylindole (DAPI) staining (Solarbio, China) lasting 10 min to investigate SAHF. The images were captured under a confocal microscope (Leica, Germany). The SAHF-positive percentage was quantified with ImageJ software.

### Immunofluorescent Staining

The sterilized cell slides were placed in a 12-well plate, along with being washed utilizing PBS. HCC cells were evenly inoculated onto the slides, and the cell density on the slides was about 40%. After treating the cells according to the experimental purpose, the cells were investigated under a microscope to achieve the ideal density. After the cells became elliptical, they were removed using curved tweezers and placed them face upward on the wet box. Following removal of PBS buffer on the cell slides, 200 μl of 4% paraformaldehyde was added, and the cells were fixed for 10 min. Then, the climbing piece was placed in 0.5% Triton penetrating solution for 20 min. PBS was aspirated, along with blocking utilizing 3% BSA lasting 1 h. The primary antibody diluent of p-ERK (1:100; 28733-1-AP; Proteintech) was slowly covered with the cell slides and kept in a refrigerator at 4°C overnight. In the dark, 200 μl of secondary antibody was dropped on the cell slide and incubated for 60 min at room temperature in the dark. Nuclei were stained by DAPI staining for 10 min. The fluorescence quencher was dropped on a clean glass slide and gently covered the cell surface of the cell slide. The capture of images was implemented utilizing a confocal microscope (Leica, Germany) and quantified with ImageJ software.

### Statistical Analysis

GraphPad Prism 8.0.1 was applied for data analysis. Data were displayed as mean ± standard deviation (SD). Comparison of two groups was evaluated with Student’s t-test. Comparison of multiple groups was conducted with one-way analysis of variance along with Bonferroni *post hoc* tests. *p* values < 0.05 were considered statistically significant. Significance was determined as follows: *p ≤ 0.05, **p ≤ 0.01; ***p ≤ 0.001; ****p ≤ 0.0001.

## Results

### Angiotensin II Type I Receptor Knockdown Alleviates Proliferation and Induces Growth Arrest for Hepatocellular Carcinoma Cells

**FIGURE 1:**
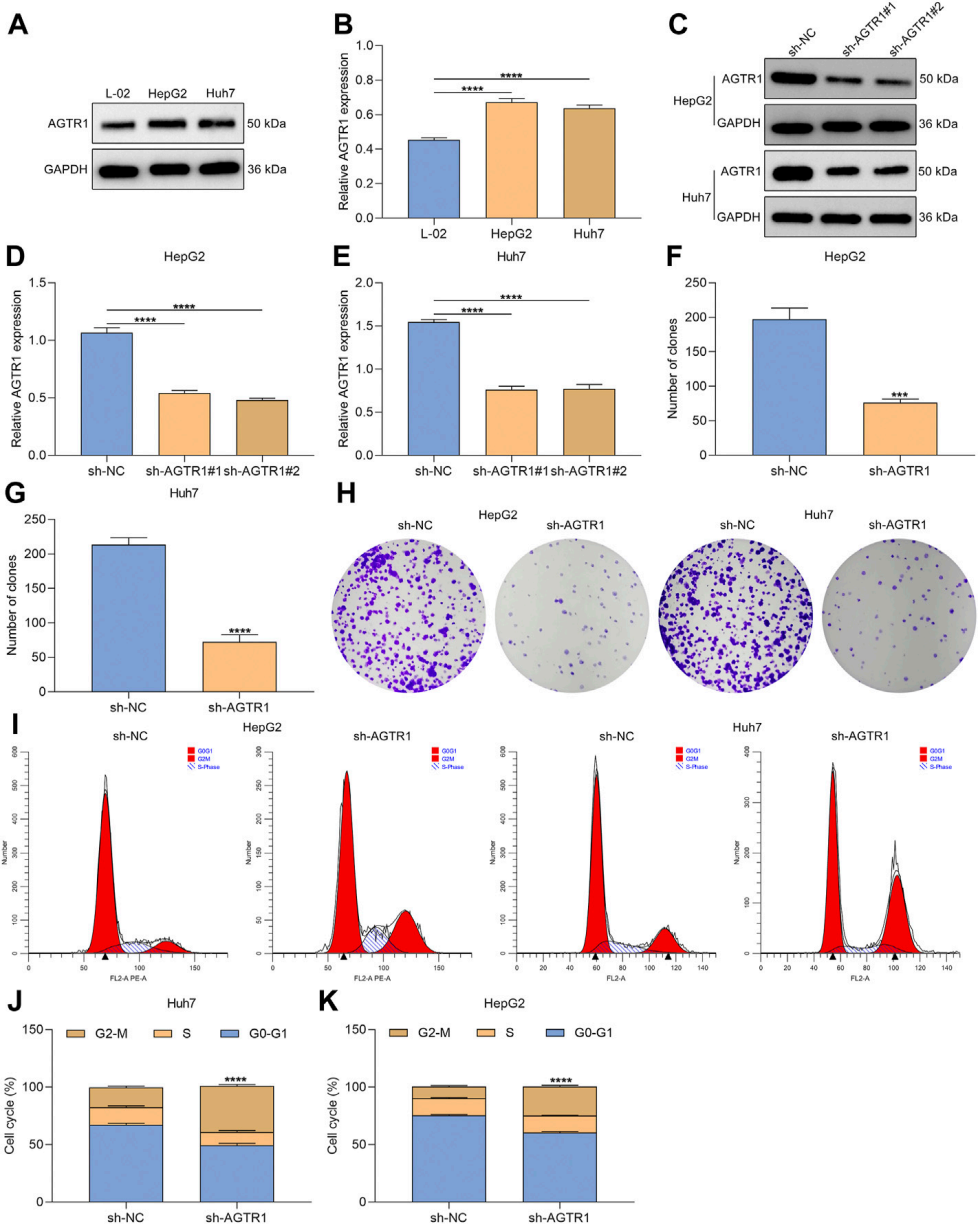
AGTR1 knockdown alleviates proliferation and induces growth arrest for HCC cells. **(A,B)** AGTR1 protein expression in L-02, HepG2 along with Huh7 cell lines. **(C–E)** AGTR1 protein expression in HepG2 and Huh7 cell lines with sh-AGTR1 lentivirus transduction. **(F–H)** Number of colonies in HCC cell lines with sh-AGTR1 lentivirus transduction. **(I–K)** Cell cycle distribution of HCC cell lines with sh-AGTR1 lentivirus transduction.

We first determined the expression of AGTR1 in human normal hepatocyte line L-02 and human HCC cell lines HepG2 and Huh7. Compared with L-02 cells, high AGTR1 expression was investigated in HCC cells (Figures 1A,B). For determining the functional role of AGTR1 in HCC, this study utilized sh-AGTR1 lentivirus for stable knockdown of AGTR1 in HepG2 and Huh7 cells. Following validation, sh-AGTR1 stably attenuated AGTR1 expression in HCC cells (Figures 1C–E). Afterward, we investigated whether suppression of AGTR1 prevented the proliferation of HCC cells. As a result, sh-AGTR1 dramatically restrained the cellular growth of HCC cells (Figures 1F–H). Cell cycle profiles were examined with flow cytometry. G2-M cell phase arrest was triggered by sh-AGTR1 (Figures 1I–K). Thus, suppression of AGTR1 enabled to alleviate proliferation and aggravate growth arrest for HCC cells.

### Angiotensin II Type I Receptor Knockdown Triggers Cellular Senescence of Hepatocellular Carcinoma Cells *via* p53/p21 Signaling

**FIGURE 2:**
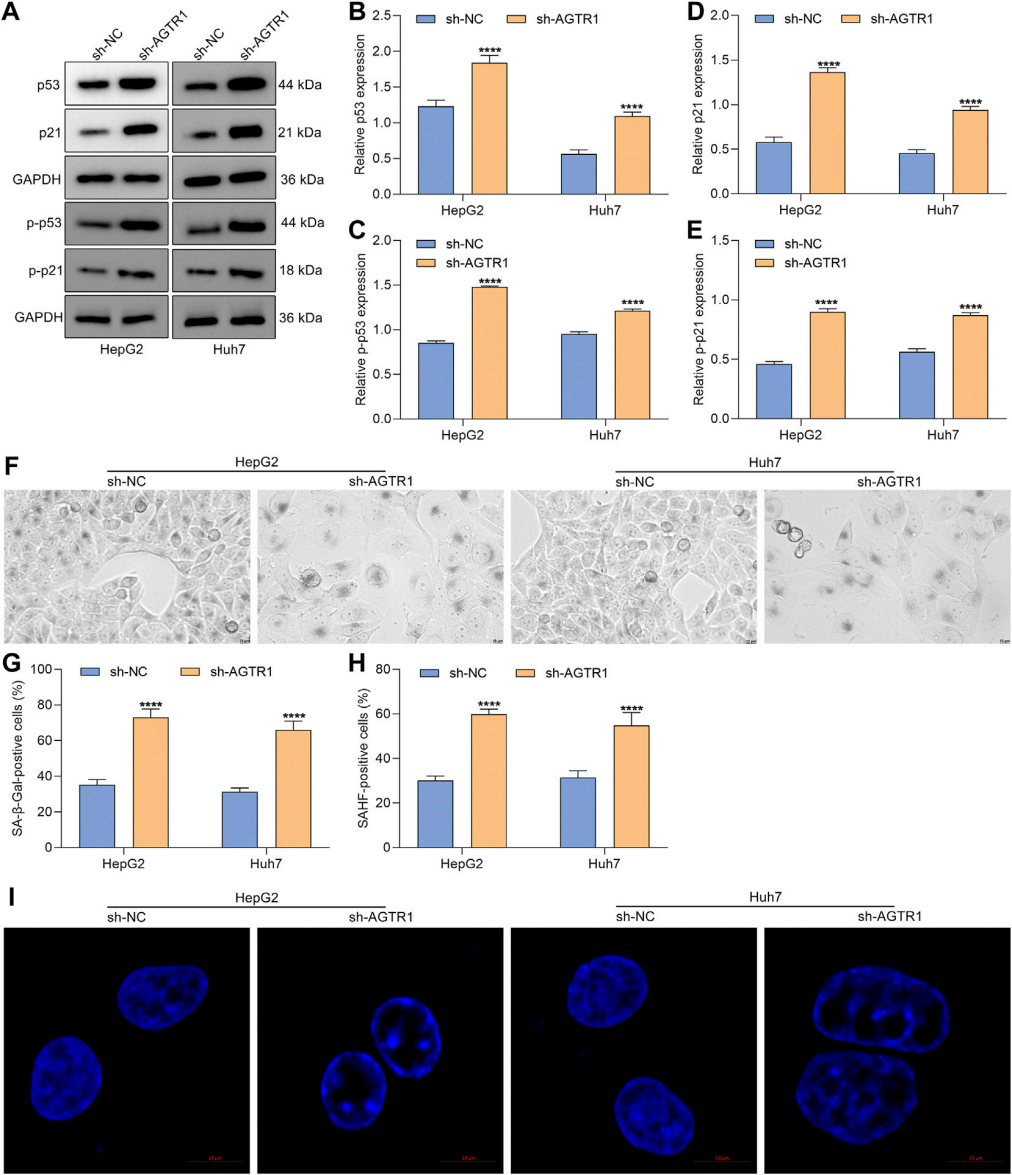
Suppression of AGTR1 triggers cellular senescence of HCC cells *via* p53/p21 signaling. **(A–E)** Expression of p53, p-p53, p21, and p-p21 in HepG2 and Huh7 cell lines with sh-AGTR1 lentivirus transduction. **(F,G)** SA-β-Gal activity of HCC cell lines with sh-AGTR1 lentivirus transduction. Scale bar, 10 μm. **(H,I)** SAHF activity of HCC cell lines with sh-AGTR1 lentivirus transduction. Scale bar, 10 μm.

Senescence is an antiproliferative mechanism, which enables inhibition of tumor development (Trayssac et al., 2021). Except for cell cycle arrest, cellular senescence was characterized by secretion of SASP and genetic alterations, etc. Here, we further determined whether AGTR1 affected cellular senescence of HCC cells. Senescence markers p53 and p21 were first examined. Under AGTR1 knockout, HepG2 and Huh7 cells, p53, p-p53, p21, and p-p21 expressions were remarkably increased (Figures 2A–E). SA-β-Gal staining that reflects enhanced lysosomal functions and lipofuscin induced by protein and lipid changes was conducted for evaluating cellular senescence. The percentages of SA-β-Gal-positive cells were markedly elevated in sh-AGTR1 lentivirus-transduction HCC cells (Figures 2F,G). We also noted that AGTR1 knockout HCC cells displayed striking phenotypic alterations (increased cell size and flattened morphology, etc.). Additionally, AGTR1 knockdown dramatically increased the percentage of SAHF-positive HCC cells (Figures 2H,I). On the basis of the obtained data, we concluded that suppression of AGTR1 was capable of triggering cellular senescence of HCC cells via p53/p21 signaling.

### Overexpressed Angiotensin II Type I Receptor Increases Proliferation and Weakens Growth Arrest for Sorafenib-Treated Hepatocellular Carcinoma Cells

**FIGURE 3:**
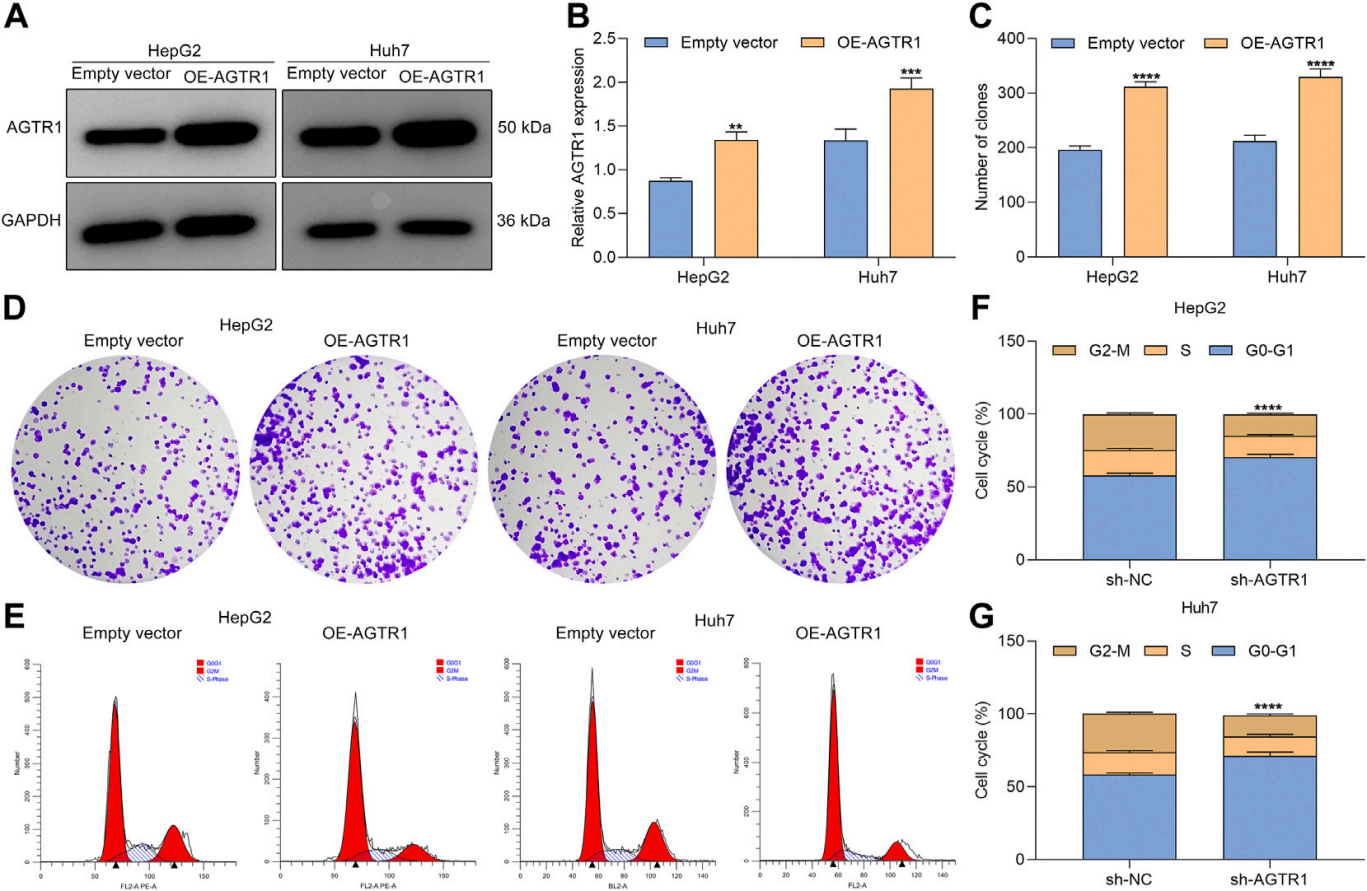
Upregulated AGTR1 increases proliferation and weakens growth arrest for sorafenib-treated HCC cells. **(A,B)** AGTR1 protein expression in sorafenib-treated HepG2 and Huh7 cell lines under AGTR1 lentivirus transduction. **(C,D)** Number of colonies of sorafenib-treated HCC cells with AGTR1 lentivirus transduction. **(E–G)** Cell cycle of sorafenib-treated HCC cells with AGTR1 lentivirus transduction.

Evidence demonstrates that cellular senescence of HCC cells can be induced by chemotherapeutics (including sorafenib) (Leung et al., 2020). To investigate whether AGTR1 affected the therapeutic effect of sorafenib in HCC cells, AGTR1-overexpressed HepG2 and Huh7 cells (Figures 3A,B) were administrated with 8 μM sorafenib lasting 4 days. We found that AGTR1 overexpression reinforced the proliferative ability of sorafenib-treated HCC cells (Figures 3C,D). Senescence-associated cell cycle arrest in cancer cells is regarded as an effective therapeutic strategy, notably those with apoptosis resistance. We investigated that G2-M phase arrest of sorafenib-exposed HCC cells was alleviated by AGTR1 overexpression (Figures 3E–G). By reason of the foregoing, AGTR1 strengthened proliferation as well as weakened growth arrest for sorafenib-treated HCC cells.

### Overexpressed Angiotensin II Type I Receptor Weakens Cellular Senescence of Sorafenib-Treated Hepatocellular Carcinoma Cells

**FIGURE 4:**
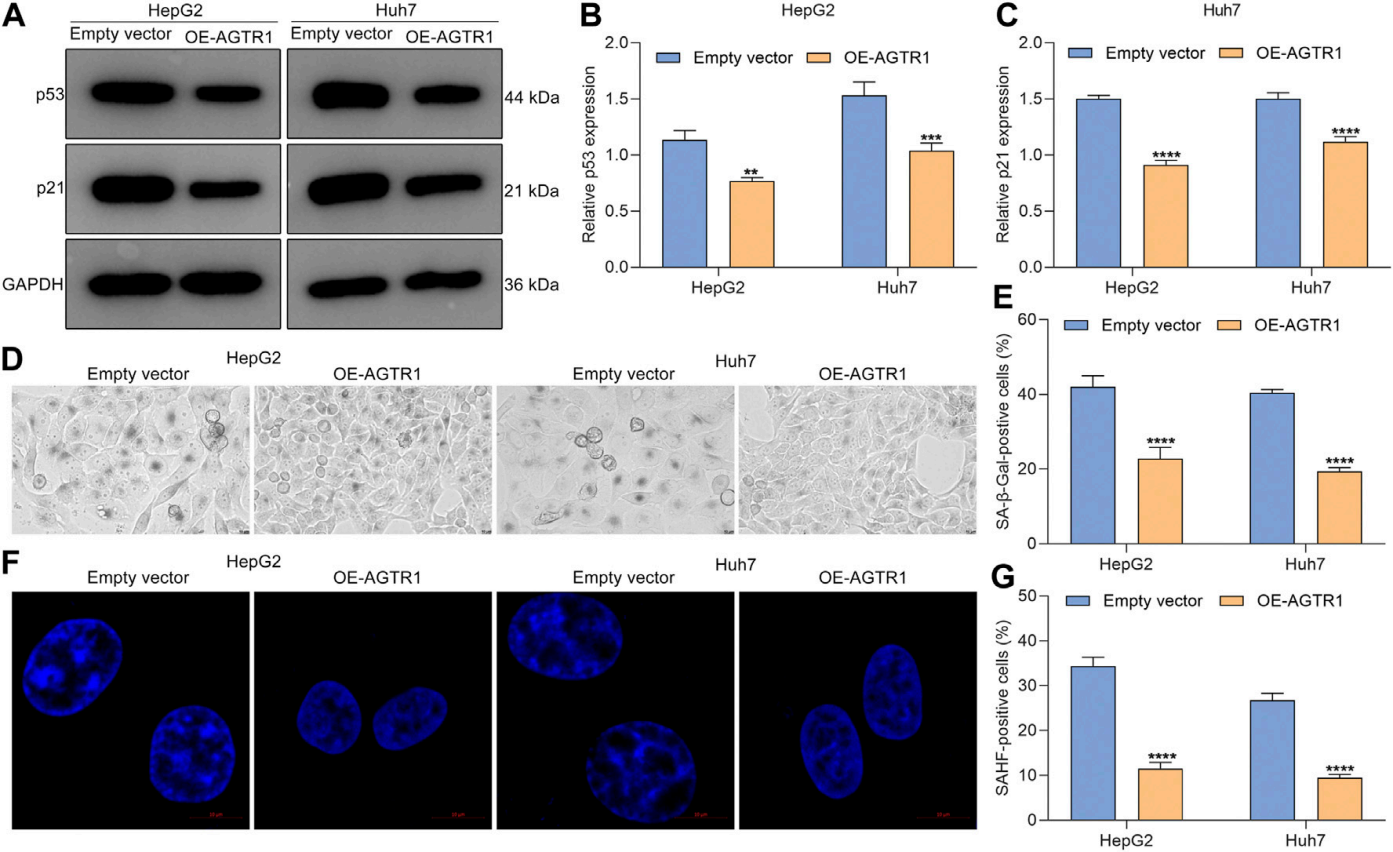
Upregulated AGTR1 weakens cellular senescence of sorafenib-treated HCC cells. **(A–C)** p53 and p21 protein expression in sorafenib-treated HepG2 along with Huh7 cell lines under AGTR1 lentivirus transduction. **(D,E)** SA-β-Gal activity of HCC cell lines with AGTR1 lentivirus transduction. Scale bar, 10 μm. **(F,G)** SAHF activity of HCC cell lines with AGTR1 lentivirus transduction. Scale bar, 10 μm.

Further analysis was conducted for determining the functional roles of AGTR1 in sorafenib-induced cellular senescence in HepG2 and Huh7 cells. The data showed that AGTR1 upregulation dramatically reduced p53 and p21 expression in sorafenib-treated HCC cells (Figures 4A–C). Moreover, the percentages of SA-β-Gal-positive sorafenib-treated HCC cells were markedly decreased by AGTR1 overexpression (Figures 4D,E). Meanwhile, upregulated AGTR1 reduced the percentage of SAHF-positive sorafenib-treated HCC cells (Figures 4F,G). On the basis of aforementioned data, we drew a conclusion that AGTR1 enabled alleviation of cellular senescence of sorafenib-treated HCC cells.

### Suppression of Angiotensin II Type I Receptor Weakens ERK Activity in Hepatocellular Carcinoma Cells

**FIGURE 5:**
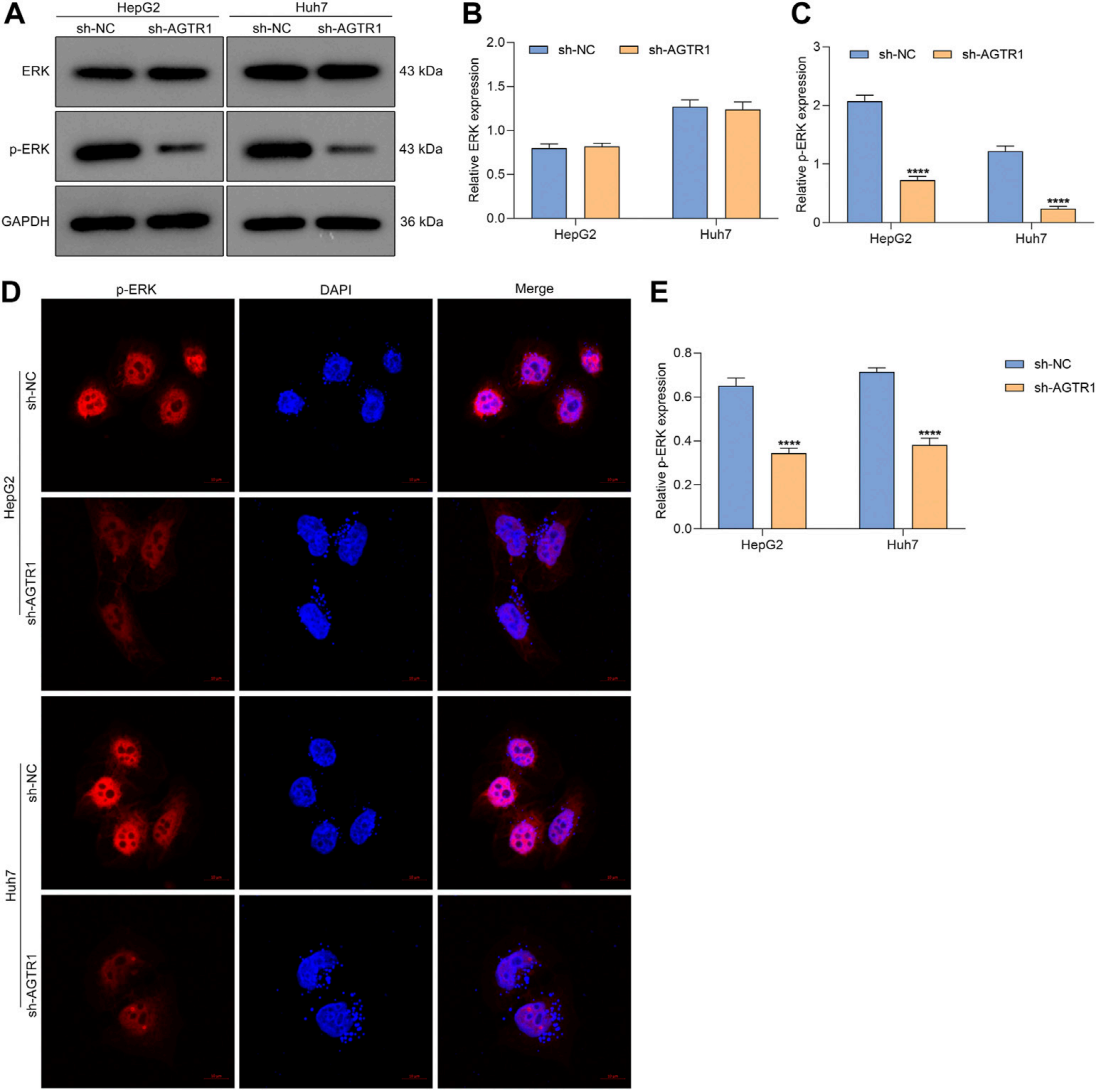
Suppression of AGTR1 weakens ERK activity in HCC cells. **(A–C)** ERK and p-ERK levels in HepG2 and Huh7 cell lines with sh-AGTR1 lentivirus transduction. **(D,E)** Immunofluorescent staining of p-ERK in HCC cell lines with sh-AGTR1 lentivirus transduction. Scale bar, 10 μm.

Evidence demonstrates the crucial role of ERK signaling in cellular senescence (Yan et al., 2019). In HepG2 and Huh7 cells, suppression of AGTR1 did not affect ERK expression but dramatically lowered p-ERK levels (Figures 5A–C). Immunofluorescent staining also confirmed the down-regulation of p-ERK in HCC cells with AGTR1 knockdown (Figures 5D,E). Hence, suppression of AGTR1 was capable of weakening ERK activity in HCC cells.

### Angiotensin II Type I Receptor Knockdown Suppresses Proliferation and Triggers Growth Arrest for Hepatocellular Carcinoma Cells Through ERK Signaling

**FIGURE 6:**
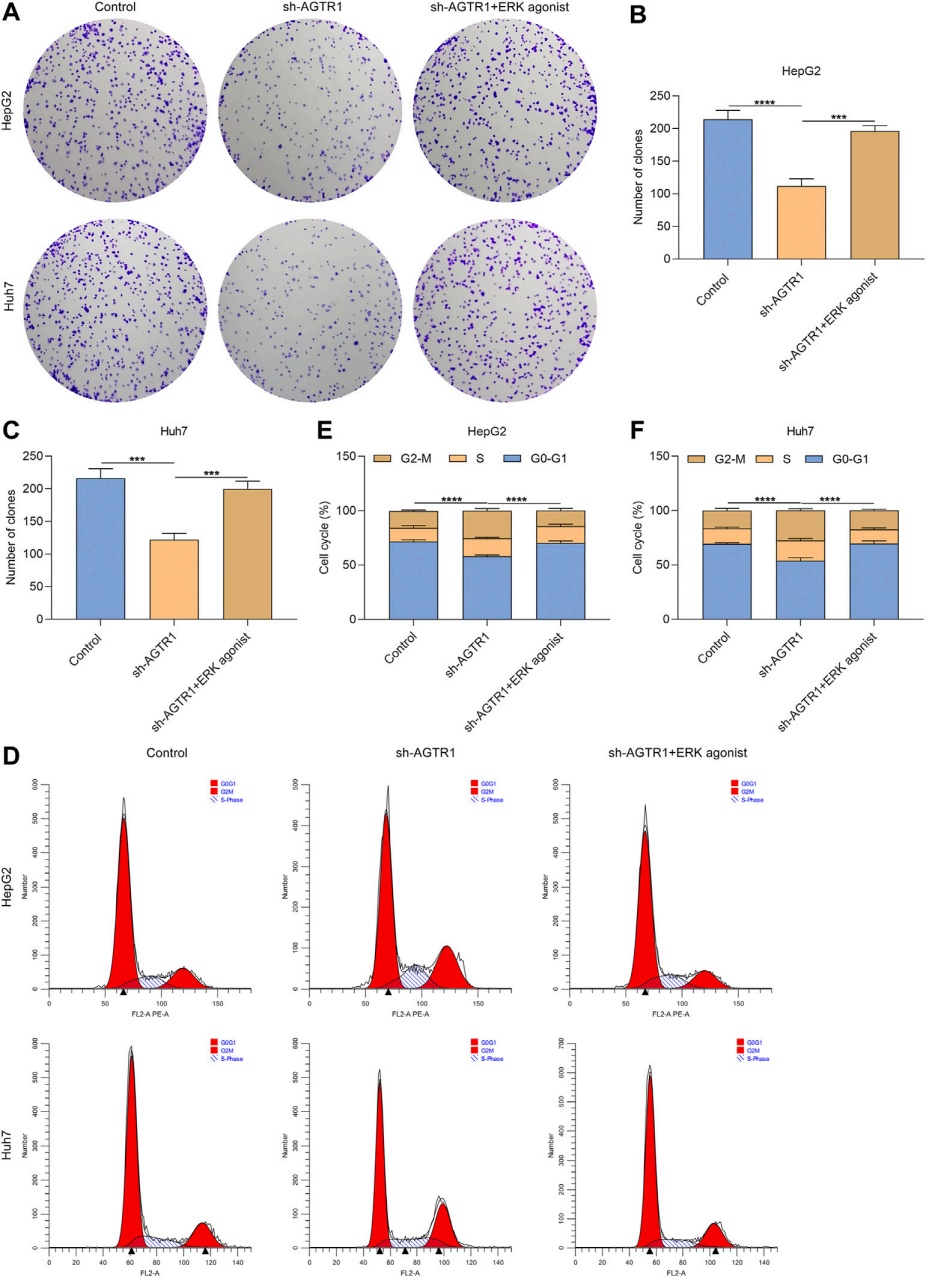
AGTR1 knockdown suppresses proliferation and triggers growth arrest for HCC cells through inactivating ERK signaling. **(A–C)** Number of colonies of HepG2 and Huh7 cell lines with sh-AGTR1 lentivirus transduction or ERK agonist EGF. **(D–F)** Cell cycle of HCC cell lines with sh-AGTR1 lentivirus transduction or ERK agonist EGF.

Further analysis was carried out for evaluating whether AGTR1 induced cellular senescence of HCC cells through modulating ERK activity. The data showed that ERK agonist EGF improved the colonies of AGTR1-knockout HepG2 and Huh7 cells (Figures 6A–C). This indicated that the proliferative capacity of AGTR1-knockout HCC cells was improved by activated ERK. Moreover, G2-M phase arrest of AGTR1-knockout HCC cells was alleviated by ERK agonist EGF (Figures 6D–F). Based on these, we concluded that suppression of AGTR1 suppressed proliferation as well as triggered growth arrest for HCC cells *via* ERK signaling.

### Angiotensin II Type I Receptor Knockdown Triggers Cellular Senescence of Hepatocellular Carcinoma Cells in an ERK-dependent Pathway

**FIGURE 7:**
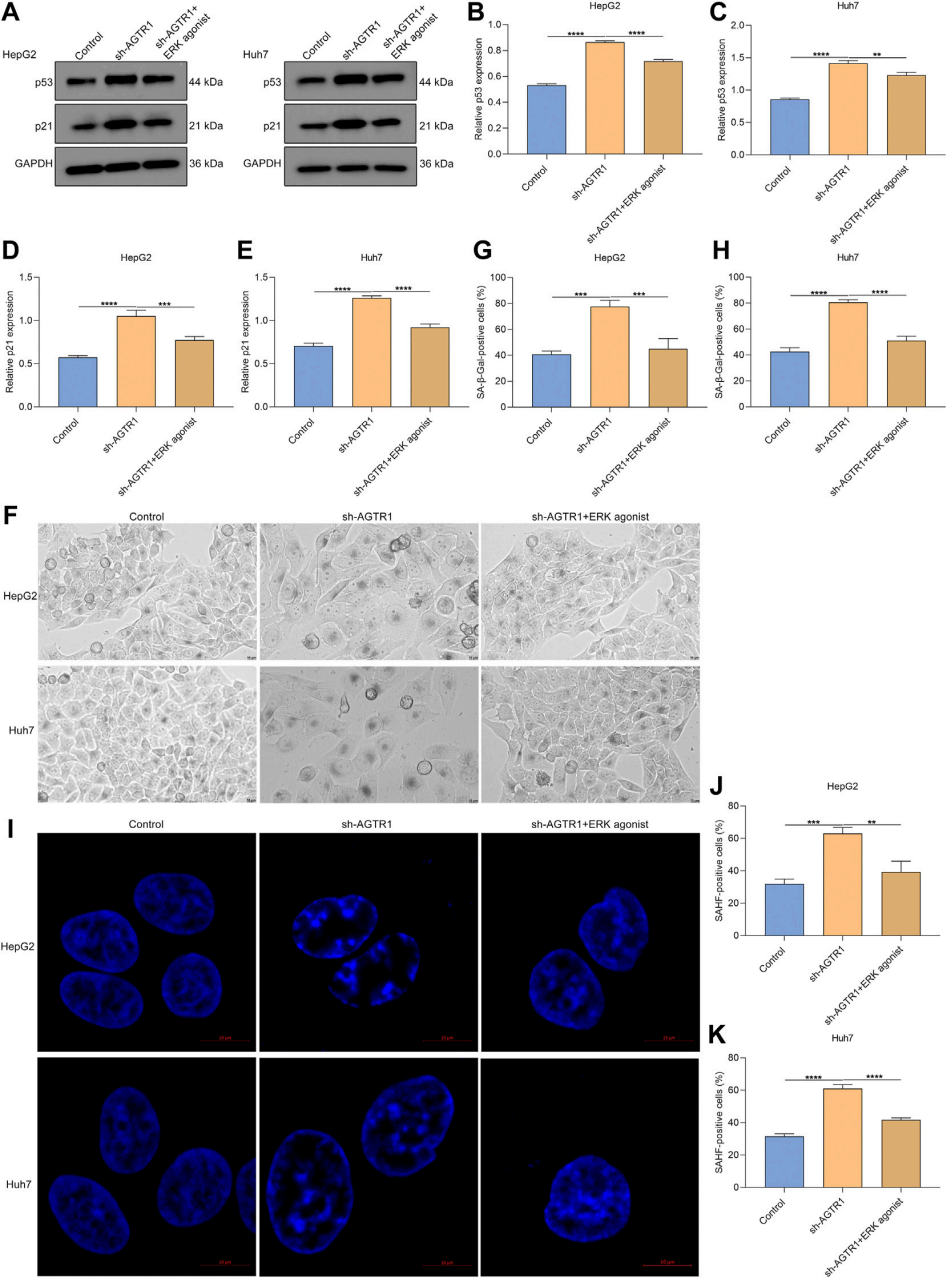
AGTR1 knockdown triggers cellular senescence of HCC cells in an ERK-dependent pathway. **(A–E)** p53 and p21 protein expressions in HepG2 along with Huh7 cell lines with sh-AGTR1 lentivirus transduction or ERK agonist EGF. **(F–H)** SA-β-Gal activity of HCC cell lines with sh-AGTR1 lentivirus transduction or ERK agonist EGF. Scale bar, 10 μm. **(I–K)** SAHF activity of HCC cell lines with sh-AGTR1 lentivirus transduction or ERK agonist EGF. Scale bar, 10 μm.

As depicted in Figures 7A–E, ERK agonist EGF dramatically improved the expression of p53 and p21 in AGTR1-knockout HepG2 and Huh7 cells. Moreover, the percentage of SA-β-Gal-positive AGTR1-knockout HCC cells was increased by ERK agonist EGF (Figures 7F–H). Meanwhile, we investigated that ERK agonist EGF markedly improved the percentage of SAHF-positive AGTR1-knockout HCC cells (Figures 7I–K). Thus, suppression of AGTR1 enabled to trigger cellular senescence of HCC cells in an ERK-dependent pathway.

## Discussion

In the present study, our evidence suggested that AGTR1 attenuated cellular senescence of HCC cells through activating ERK signaling, indicating AGTR1 as a drug target against HCC. HCC is still difficult to treat due to a lack of drugs targeting key dependencies (Gao et al., 2021), and broad-spectrum kinase antagonists (especially sorafenib) have little benefit in HCC patients (Kelley et al., 2021; Ren et al., 2021; Wen et al., 2021). Experimental evidence suggests that sorafenib enables to facilitate cellular apoptosis and senescence and mitigates angiogenesis as well as suppresses proliferation in cancer cells (Ding et al., 2021; Ryoo et al., 2021). Overexpressed AGTR1 weakened sorafenib-induced cellular senescence in HCC cells, indicating that AGTR1 suppression and sorafenib as a potential combination therapy might synergistically inhibit HCC progression.

Upregulated AGTR1 was found in human HCC cell lines HepG2 and Huh7 in comparison to human normal hepatocyte line L-02. Previous studies have demonstrated the upregulation of AGTR1 in other cancer types (breast cancer, etc.) (Qiao et al., 2021). Lentivirus vector-mediated sh-AGTR1 was utilized for stably silencing AGTR1 in HCC cells. Our data demonstrated the inhibitory effect of AGTR1 knockdown on the proliferative ability of HCC cells. Additionally, AGTR1-knockout HCC cells presented G2-M cell phase arrest. Senescence is a status in which stable cell cycle arrest is triggered by intrinsic or extrinsic damage (Innes et al., 2018). Senescent cells have the key features of persistent cell cycle arrest, elevated lysosomal contents called SA-β-Gal activity, markedly increased expression of cyclin-dependent kinase inhibitors, persistent DNA damage response, the abnormal changes in the structure of condensed chromatin called SAHF, and secretion of SASP (Zhang et al., 2021). Evidence suggests that cellular senescence is a key tumor suppression mechanism, which may prevent oncogenic activation and genetic instability as well as damaged cells. Inducing cellular senescence of tumor cells is an underlying mechanism by which cancer therapies exert antitumor activity (Birch and Gil, 2020). The tumor suppressor p53 is capable of restricting malignant transformation through inducing a cell-autonomous program of cell cycle arrest as well as apoptosis (Wang et al., 2021). Additionally, p53 enables facilitation of cellular senescence, involving stable cell cycle arrest as well as secretion of factors that alter the tissue microenvironment (Lujambio et al., 2013). In HCC, cellular senescence is primarily controlled by p53-dependent or -independent signaling (Xiang et al., 2021). Understanding the molecular signaling that mediates cancer cell senescence enables to yield new insights into guiding the discovery of unique antitumor agents as well as molecular biomarkers (Qiu et al., 2020). In our study, cellular senescence of HCC cells was triggered by AGTR1 knockdown in accordance with weakened proliferative ability, G2-M phase arrest, and increased expression of p53 and p21 as well as the proportions of SA-β-Gal- and SAHF-positive cells, indicating that AGTR1 attenuated tumor cellular senescence during HCC progression.

Senescence occurs following chemotherapies (sorafenib, etc.), called therapy-induced senescence through inducing DNA double-strand breaks (Tang et al., 2020). We found that overexpressed AGTR1 heightened proliferation as well as alleviated G2-M cell phase arrest for sorafenib-treated HCC cells. Additionally, AGTR1 upregulation decreased p53 and p21 expressions as well as the proportions of SA-β-gal- and SAHF-positive cells for sorafenib-exposed HCC cells. Several therapeutic options, notably sorafenib, offer only modest survival benefits to patients with HCC (Lyu et al., 2022; Öcal et al., 2022). A combination of AGTR1 suppression and sorafenib might be a potential therapeutic regimen to synergistically restrain HCC progression.

The ERK signaling pathway exerts a key in nearly all cellular functions (especially proliferation and senescence) (Berger et al., 2017). Evidence indicates that AGTR1 mediates ERK activity in prostate cancer (Zhang D et al., 2019). In HCC cells, suppression of AGTR1 enabled weakening of ERK activity. ERK agonist EGF improved the proliferative ability as well as decreased G2-M cell phase arrest in HCC cells. Additionally, the expression of p53 and p21 as well as the proportions of SA-β-gal- and SAHF-positive cells for AGTR1-knockout HCC cells was ameliorated by ERK agonist EGF in HCC cells. Hence, suppression of AGTR1 induced cellular senescence in HCC through inactivating ERK signaling. Despite this, the effect and mechanisms of AGTR1 inhibition on cellular senescence of HCC should be investigated *in vivo* in our future studies.

## Conclusion

Our study offered comprehensive evidence that AGTR1 exerted a key role in cellular senescence of HCC cells. Following exposure to sorafenib, overexpressed AGTR1 enabled to resist cellular senescence of HCC. Additionally, AGTR1 inhibition induced cellular senescence of HCC in an ERK-dependent pathway. Collectively, our findings highlighted the therapeutic implications of AGTR1 in HCC patients.

## Data Availability

The original contributions presented in the study are included in the article/Supplementary Material; further inquiries can be directed to the corresponding author.

## Abbreviations

HCC, hepatocellular carcinoma; SASP, senescence-associated secretory phenotype; AGTR1, angiotensin II type I receptor; CXCR4, C-X-C chemokine receptor type 4; SDF-1α, stromal cell-derived factor-1α; RPMI-1640, Roswell Park Memorial Institute media 1640; shRNA, short hairpin RNA; SDS-PAGE, sodium dodecyl sulfate-polyacrylamide gel electrophoresis; SA-β-gal, senescence-associated β-galactosidase; SAHF, senescence-associated heterochromatin foci; DAPI, 4’,6-diamidino-2-phenylindole; SD, standard deviation.
